# Bioprospecting microwave-alkaline hydrolysate cocktail of defatted soybean meal and jackfruit peel biomass as carrier additive of molasses-alginate-bead biofertilizer

**DOI:** 10.1038/s41598-021-02170-w

**Published:** 2022-01-07

**Authors:** Muhamad Aidilfitri Mohamad Roslan, Zulfazli M. Sobri, Ali Tan Kee Zuan, Sim Choon Cheak, Nor Aini Abdul Rahman

**Affiliations:** 1 Department of Bioprocess Technology, Faculty of Biotechnology and Biomolecular Sciences, Universiti Putra Malaysia, 43400 Serdang, Selangor Malaysia; 2 Department of Land Management, Faculty of Agriculture, Universiti Putra Malaysia, 43400 Serdang, Selangor Malaysia; 3 R&D Center, Sime Darby Plantation Research Sdn. Bhd., 42960 Carey Island, Selangor Malaysia

**Keywords:** Environmental biotechnology, Industrial microbiology

## Abstract

The extraction of soluble hydrolysate protein and sugar from a biomass cocktail of defatted soybean meal (DSM) and jackfruit peel (JP) was examined using microwave-alkaline hydrolysis by varying the NaOH concentrations (0.04–0.11 M) and residence times (2–11 min). Based on the central composite design, the optimized parameters were achieved at 0.084 M NaOH concentration (100 mL), for 8.7 min at 300 W microwave power level to obtain the highest protein (5.31 mg/mL) and sugar concentrations (8.07 mg/mL) with > 75% recovery. Both raw and detoxified hydrolysate (using activated carbon) were correspondingly biocompatible with *Enterobacter hormaechei* strain 40a (*P* > 0.05) resulting in maximal cell counts of > 10 log CFU/mL. The optimized hydrolysate was prepared as an additive in molasses-alginate bead encapsulation of strain 40a. Further evaluation on phosphate and potassium solubilization performance of the encapsulated strain 40a exhibited comparable results with those of free cell counterpart (*P* > 0.05). The DSM-JP hydrolysate cocktail holds potential as a carrier additive of encapsulated-cell bead biofertilizers in order to sustain bacterial cell quality and consequently improve crop growth and productivity.

## Introduction

The application of targeted plant-beneficial microbes with multifunctional capabilities either by single species or consortia is an excellent tool to boost crop health and productivity^1^. Plant growth is influenced by a variety of soil microorganisms, including root endophytes, mycorrhizal fungi, plant growth-promoting bacteria, rhizobia, and phosphate (P) and potassium (K) solubilizers, through direct and indirect mechanisms, even in stressful environments^2–5^. While studies on the isolation and characterization of plant-beneficial microbes are aplenty in the scientific literature, only a handful have made it to the commercial market. Most commercial bioinoculants lack efficiency under field conditions as compared to preliminary trials demonstrated in the laboratory and/or greenhouse^6^ due to inadequate and/or poor quality formulation e.g., low carrier biocompatibility and stability^7,8^. Collectively, these drawbacks create a serious impediment to efficient practice and advancement of bioinoculants on a massive scale.

Bacterial encapsulation technology has the potential to bridge the gap between the effective use of bioinoculants and the practicality of bioinoculants as biofertilizers in the agriculture industry. Encapsulation technology offers a unique delivery system that improves the application of soil bioinoculants while allowing tailor-made formulation of carriers in particular to their targeted functions^9,10^. This technology essentially provides a protective barrier that allows a controlled release of microorganisms and maintains their functional ability in the long run^11^. Sodium alginate is one of the encapsulation materials that has gone through extensive research since it can produce microbeads instantly in the presence of calcium as cross-linker or polyvalent cation^9^. The capacity of the alginate matrix to establish a favourable micromilieu for bacterial cells by feeding vital nutrient sources permits cell metabolic activity to be maintained for longer periods^12^.

To this end, various types of additives have been employed in bioencapsulation systems to serve as bulking agents and/or stabilizers, other than to preserve and nourish the immobilized cells or spores^13^. Hydrolysis derivatives such as protein and sugar hydrolysates from chemical, thermal and enzymatic treatments of plant biomass have been reported to boost plant nutrient uptake and soil microbial activity while feeding supplementary nutrients for the encapsulated cells as well as the soil rhizosphere^14–17^. For instance, Vejan et al.^18^ stated that encapsulation of *Bacillus salmalaya* using chitosan-alginate-protein (brown rice) had resulted in 99.7% and 89.3% encapsulation efficacy of pre- and post-freeze-drying, respectively. Additionally, existing research recognizes the critical role played by sugars (e.g., sucrose, trehalose, glucose, etc.) to facilitate cellular protection against osmotic pressure fluctuations, particularly during the drying process^19^. This has been demonstrated by Mishra et al.^17^ who recorded 100% conidial germination and 78% conidial viability by the encapsulated *Beauveria bassiana* formulated using skim milk, polyvinylpyrrolidone K-90, and glucose as additives even after 12 months of storage at 30 °C.

Bioprospecting of agricultural biomass holds great promise for recovering low-cost, natural proteins and sugars. Defatted soybean meal (DSM), a by-product of soybean oil extraction is widely used as animal feeds since it is high in protein, essential amino acids, and other micronutrients^20^. DSM protein has been recently repurposed for nisin production, fumaric acid production, adhesive formulation, and copolymer fertilizer^21–24^. Jackfruit peel (JP) is one of the lignocellulosic feedstocks which barely receive commercial importance and valorization prospect. Although there have been a few investigations to recover heteropolysaccharide pectin and nanocellulose crystals from its fibrous rinds^25–27^, sugar reprocessing from its hydrolyzed polysaccharide components is scarce and mostly limited to biofuel production^28^. Blackstrap molasses, a viscous, dark syrup produced abundantly as a by-product of the sugar beet and sugarcane refinery industries, is another example of sugar-rich industrial waste^29^. It is a renewable resource that contains up to 75% of sugars and is high in organic acids (e.g., humic acid, fulvic acid, etc.) which are beneficial for plant growth^30^.

Attempts on natural protein and sugar recovery from agricultural waste mostly involve acidic or alkaline pretreatments since they offer several benefits including the convenience of operation and low cost. Compared to acid pretreatments, mild alkaline hydrolysis results in fewer production of inhibitory compounds (e.g., hydroxy acids, dicarboxylic acids, and phenolic compounds, etc.) at the expense of less hemicellulose breakdown^31^. Therefore, recent studies have combined thermal and alkaline treatments to optimize the hydrolysis efficiency such as those described by Cheong et al. and Lee et al.^32,33^ who incorporated microwave-alkaline hydrolysis (MAH) to solubilize recalcitrant chicken feathers. Considering the pros and cons of the hydrolysis strategies, the present study aimed to investigate the effects of MAH treatment on the DSM and JP biomass cocktail in order to optimize the concentration of soluble protein and sugar in the hydrolysate. The biocompatibility of the optimized hydrolysate as a growth medium of *Enterobacter hormaechei* 40a was evaluated prior to encapsulation using molasses-alginate bead formulation. The performance of free cell and encapsulated strain 40a in P and K solubilization was then compared to verify its feasibility.

## Results and discussion

### Optimization of microwave-alkaline hydrolysis

The nutrient analysis of both DSM and JP, as well as molasses, were broken down into several main nutritional components as shown in Table 1. More than 50% nutrient fraction of DSM consisted of protein while more than two-thirds of JP comprised of carbohydrates. Both substrates are excellent nitrogen and carbon sources respectively which fit the nutrient requirement for strain 40a proliferation. In addition, they were rich in trace minerals (Supplementary information) especially phosphorus, potassium, calcium, magnesium, and sodium. To obtain a homogenized liquid formulation, both DSM and JP were hydrolyzed concurrently through the MAH technique to extract a high amount of soluble proteins and sugars. Previous reports have successfully demonstrated the same MAH technique to extract crude proteins from chicken feathers^32,33^. To the best of our knowledge, the extraction of soluble protein and sugar from the DSM-JP cocktail has yet to be reported particularly through MAH treatment.

**Table 1:** Nutrient fraction (g/100 g) of defatted soybean meal (DSM) and jackfruit peel (DSM).

Sample	Protein	Total fat	Total carbohydrate	Ash	Moisture
DSM	51.17 ± 1.53	0.07 ± 0.01	31.56 ± 1.53	6.80 ± 0.02	10.41 ± 0.01
JP	8.11 ± 0.04	1.65 ± 0.08	75.39 ± 0.39	6.19 ± 0.07	8.66 ± 0.2
Molasses	1.17 ± 0.01	< 0.01	62.07 ± 0.13	4.03 ± 0.03	30.40 ± 0.12

Values were stated in mean ± standard deviation (n = 3).

The parameters described by Cheong et al.^32^, were adopted in the present central composite design (CCD) to achieve the optimal protein and sugar concentration of hydrolysate. The experimental setup and the results of CCD were presented in Table 2. It was observed from the table that the protein concentration of hydrolysate varied from 1.22 (run 10) to 1.92 mg/mL (run 7) while the sugar concentration ranged from 3.02 (run 3) to 4.88 mg/mL (run 7). The regression analysis of the full second-order polynomial model was shown in Table 3. The high F-value (protein = 82.2, sugar = 64.5) of both models suggested that they were exceptionally significant that the probability of noise interference was only 0.01%. The relatively high value of the determination coefficient, R^2^ (protein = 0.9833, sugar = 0.9788) revealed that both models fitted to the experimental results excellently which provides a good estimation of protein and sugar concentration (*P* < 0.05).

**Table 2:** Central composite design of microwave-alkaline hydrolysis and the corresponding responses.

Run	NaOH concentration (M)	Residence time (min)	Protein concentration (mg/mL)	Sugar concentration (mg/mL)
1	0.075	11.6569	5.12	7.49
2	0.1	2	4.72	6.23
3	0.075	0.343146	4.59	6.12
4	0.05	10	5.09	6.47
5	0.110355	6	4.83	7.26
6	0.075	6	5.12	7.91
7	0.075	6	5.22	7.98
8	0.075	6	5.18	7.78
9	0.075	6	5.22	7.69
10	0.05	2	4.52	6.65
11	0.075	6	5.20	7.87
12	0.0396447	6	4.67	6.15
13	0.1	10	5.03	7.82

**Table 3:** Analysis of variance (ANOVA) for the response surface quadratic model of MAH of DSM-JP on protein and sugar concentration of hydrolysate.

Source	Protein concentration					Sugar concentration				
	SS	df	MS	F-value	*P*-value	SS	df	MS	F-value	*P*-value
Model	0.79	5	0.16	82.20	< 0.0001	6.50	5	1.30	64.50	< 0.0001
A-NaOH concentration	0.02	1	0.02	8.96	0.0201	0.78	1	0.78	38.79	0.0004
B-Residence time	0.34	1	0.34	174.32	< 0.0001	1.41	1	1.41	69.83	< 0.0001
AB	0.02	1	0.02	8.57	0.0221	0.78	1	0.78	38.84	0.0004
A^2^	0.30	1	0.30	156.20	< 0.0001	2.17	1	2.17	107.83	< 0.0001
B^2^	0.17	1	0.17	90.16	< 0.0001	1.81	1	1.81	89.90	< 0.0001
Residual	0.01	7	0.002			0.14	7	0.02		
Lack of Fit	0.006	3	0.002	1.06	0.4578	0.09	3	0.03	2.26	0.2231
Pure Error	0.008	4	0.002			0.05	4	0.01		
Cor Total	0.806	12				6.64	12			

*SS* sum of squares, *df* degree of freedom, *MS* mean square.

Protein concentration: R^2^ = 0.9833, R^2^_adj_ = 0.9713, R^2^_pred_ = 0.9326, adequate precision = 22.8576.

Sugar concentration: R^2^ = 0.9788, R^2^_adj_ = 0.9636, R^2^_pred_ = 0.8926, adequate precision = 17.8715.

**Figure 1:**
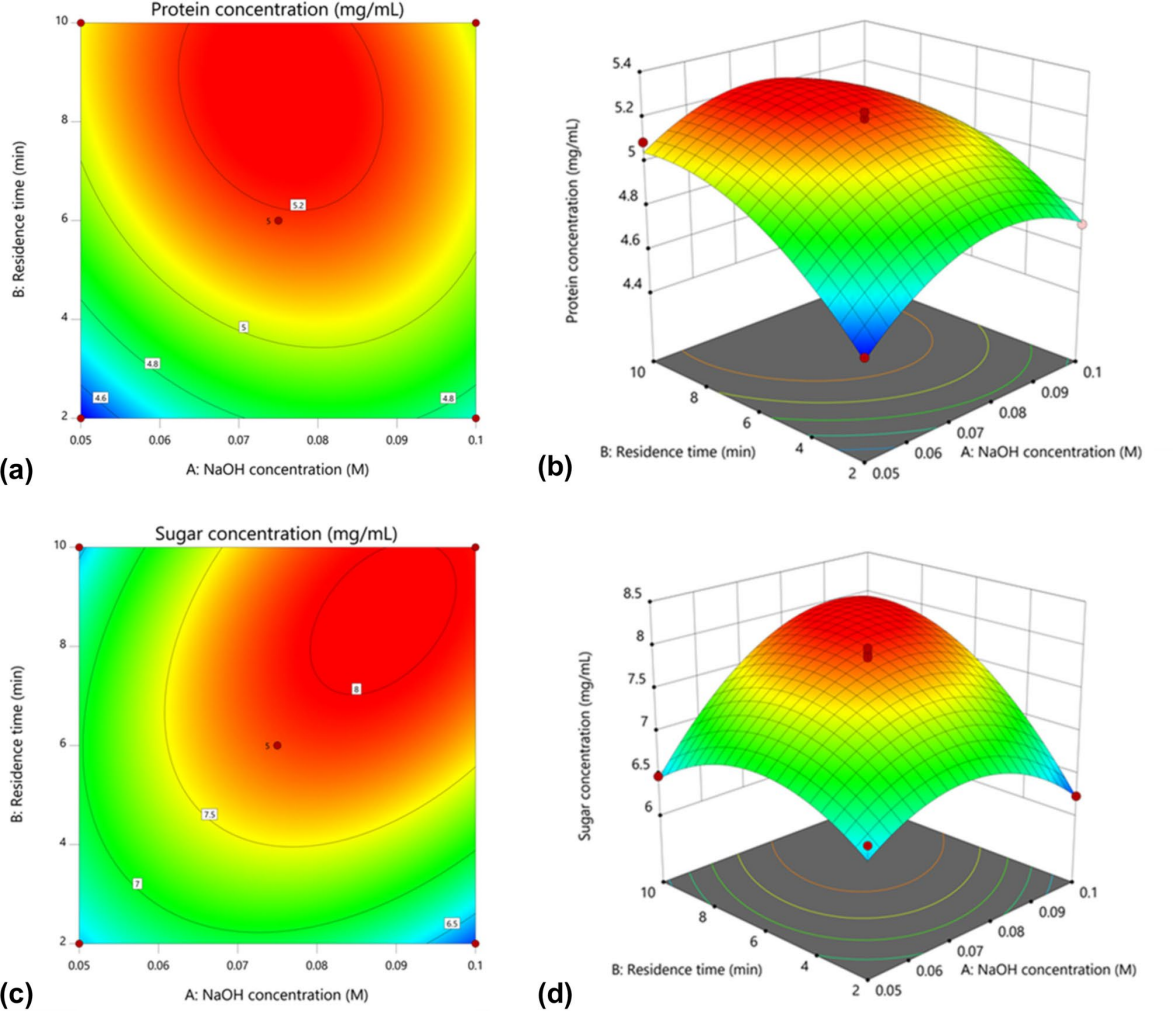
Contour (**a**,**c**) and 3D (**b**,**d**) plots of the interaction between NaOH concentration and residence time in the MAH cocktail of DSM and JP on protein and sugar concentration of hydrolysate. The graphs were visualized in DOE software (v 11; Stat-Ease, Inc., MN, USA; www.statease.com).

In both cases, all linear and quadratic functions i.e., A, B, A^2^, and B^2^ were observed as significant model terms (*P* < 0.05), implying that both NaOH concentration and residence time played significant roles in determining the protein and sugar concentration of hydrolysate in MAH protocol. Likewise, the AB combination showed a significant interaction (*P* < 0.05) for both responses, thus verifying the importance of optimizing both parameters, A and B simultaneously. The polynomial models for protein (Y_P_) and sugar (Y_S_) concentration for the MAH cocktail of DSM and JP were regressed by considering the significant terms and thereafter expressed in Eqs. (1) and (2).

1 $$Y_{P} = 5.19 + 0.0465A + { }0.205B - 0.0643AB - 0.2081A^{2} - 0.1581B^{2}$$

2 $$Y_{S} = 7.84 + 0.3126A + { }0.4194B + 0.4423AB - 0.5588A^{2} - 0.5103B^{2}$$

where Y_P_ and Y_S_ were the coded terms for protein and sugar concentration of hydrolysate, respectively. The regression models (Eqs. (1) and (2)) were used to predict the range of protein and sugar concentration dynamics for various levels of the selected variables. Consistent with the model equations, the maximum protein and sugar concentration was demonstrated by the contour and 3D plots shown in Fig. 1 by varying the parameter levels that were priorly determined by the CCD.

### Post-analysis of quadratic models

**Figure 2:**
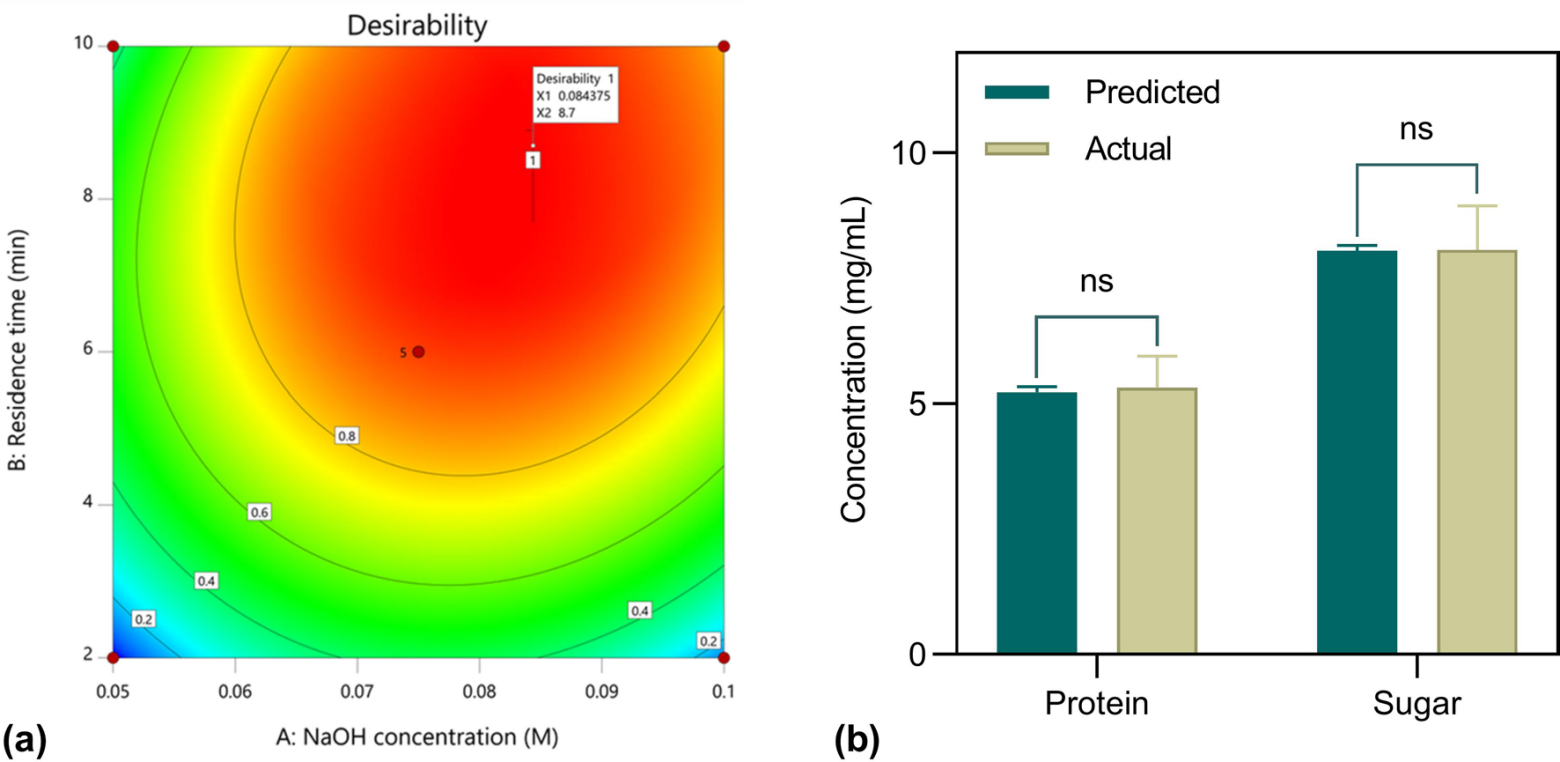
Validation of the quadratic optimization model: contour plot of quadratic model desirability prediction (**a**); experimental runs of MAH of DSM-JP cocktail based on the predicted optimum parameters (**b**). Data presented are the means ± standard deviations from three independent experiments (Student’s *t*-test: *P < 0.05; **P < 0.01; ns *P* > 0.05). The graphs were visualized in DOE software (v 11; Stat-Ease, Inc., MN, USA; www.statease.com) and GraphPad PRISM software (v 8.02; GraphPad, Inc., MN, USA; www.graphpad.com).

To validate the model adequacy and investigate the reproducibility of the generated quadratic models of the CCD, the MAH procedure was recreated using the optimized parameters with a high desirability value (1.00) to achieve hydrolysate with maximum soluble protein and sugar concentration (Fig. 2). The results were interpreted by comparing the actual data and the predicted values of the previous quadratic models as illustrated in Fig. 2b. The figure shows that the protein and sugar concentration of the actual data was 5.31 and 8.07 mg/mL, respectively. These results accord with the predicted value of protein and sugar concentration i.e., 5.23 and 8.05 mg/mL, respectively, with no significant difference (*P* > 0.05) observed than the actual value. Interestingly, both responses showed slightly higher concentration up to 1.5% than the predicted value.

These results provide further support that the newly developed model was accurate and reliable to achieve maximum yield of protein and sugar extraction from the DSM-JP cocktail hydrolysis. Therefore, the optimized parameters for the MAH treatment of 2% (w/v) DSM-JP cocktail were finalized at NaOH concentration 0.084 M (100 mL), 8.7 min residence time at 300 W microwave power-level. The hydrolysate produced under the optimized parameters was used for the subsequent experiments.

### Soluble protein and total sugar recovery

In general, DSM consists of 48–54% crude protein which largely constitutes essential amino acids such as arginine, methionine, and lysine^20,34^. The protein constituents in DSM vary and mainly depend upon the processing at the crushing plant and removal of the hulls^35^. In the present study, the total protein content of DSM and JP combined was 0.296 g/g dry mass (Table 4). With the total soluble protein yield of 531.14 mg/100 mL, the optimized MAH protocol achieved a high protein recovery of 0.896 g/g protein and 0.265 g/g dry mass. As opposed to a previous report, the protein extraction via microwave irradiation alone with water as a solvent yielded only 0.782 g/g protein even with a high (10%) biomass loading concentration^36^. The protein recovery per dry mass DSM-JP by the optimized MAH conditions was also superior to hydrothermal (water-based) treatment as reported by Watchararuji et al.^37^ who obtained a maximum of 0.205 g/g DSM.

**Table 4:** Protein and sugar recovery from MAH of DSM-JP cocktail.

Extract	Total content, g/g dry mass	Amount extracted, mg/100 ml	Recovery, g/g protein or sugar	Recovery, g/g dry mass
Protein	0.296	531.14	0.896	0.265
Sugar	0.535	806.52	0.754	0.403

The observed improvement of protein recovery in the present study might be attributed to the use of NaOH solvent in the MAH treatment. Since NaOH is more polar than water, it is more effective at dissolving complex protein molecules in the biomass. For instance, Lu et al.^38^ managed to obtain a maximum 0.723 g/g protein recovery from microalgal residue when a concentrated 7.9% NaOH solvent was used in the extraction. In addition, the MAH technique used in this investigation employed a low solid to liquid ratio (1:50 w/v). This allowed a great driving force of mass transfer during hydrolysis in which hindered biomass clumping, thus enhanced biomass dispersion, and improved protein and sugar extraction^38^. These results corroborate the findings of Cheong et al.^32^, where they successfully achieved more than 70% protein recovery using a low ratio of chicken feather biomass to NaOH (1:50 w/v) in the microwave-savinase hydrolysis experiments.

In terms of sugar extraction, the total sugar content of DSM-JP was almost double the protein content, which was 0.535 g/g dry mass (Table 4). With the total soluble sugar yield of 806.52 mg/100 mL, the optimized MAH protocol exhibited a high sugar recovery of 0.754 g/g sugar and 0.403 g/g dry mass. The high sugar content in the hydrolysate might be contributed by the degradation of various complex carbohydrate macromolecules in the JP biomass i.e., pectin—7.52% (heteropolysaccharide), cellulose—27.75%, and starch—4.12% (homopolysaccharide)^39^. Pectin is degraded into rhamnose, uronic acids, and neutral sugars e.g., d-galactose, d-glucose, or l-arabinose in alkaline pH through alkaline demethoxylation (saponification) and depolymerization (*β*-elimination) particularly when assisted with heat radiation^40^. Additionally, *β*-elimination produces unsaturated uronides, which leads to the occurrence of non-enzymatic browning^41^. These mechanisms might explain the colour transformation of the DSM-JP hydrolysate solution from dull yellowish-brown into dark brownish red during the MAH process.

Apart from the other popular physicochemical treatments used in many studies to accomplish successful protein and sugar hydrolysis, such as hydrothermal, acid, and oxidative treatments, the alkaline treatment also facilitates access to the most recalcitrant structures of polysaccharides and their reactivity. On the one hand, they can remove hemicellulose, pectin, proteins, and extracts, while also shortening cellulose fibers and increasing crystallinity^42^. Most of all, the microwave heat irradiation used in this investigation, loosened the cell wall matrix, causing parenchymal cells to sever, resulting in skin tissue opening^43^. As a result, the NaOH solvent was able to permeate the skin tissues which led to greater solvent-tissue contact, hence improving the extraction efficacy.

### Alterations of the chemical structure of raw and hydrolyzed DSM-JP

**Figure 3:**
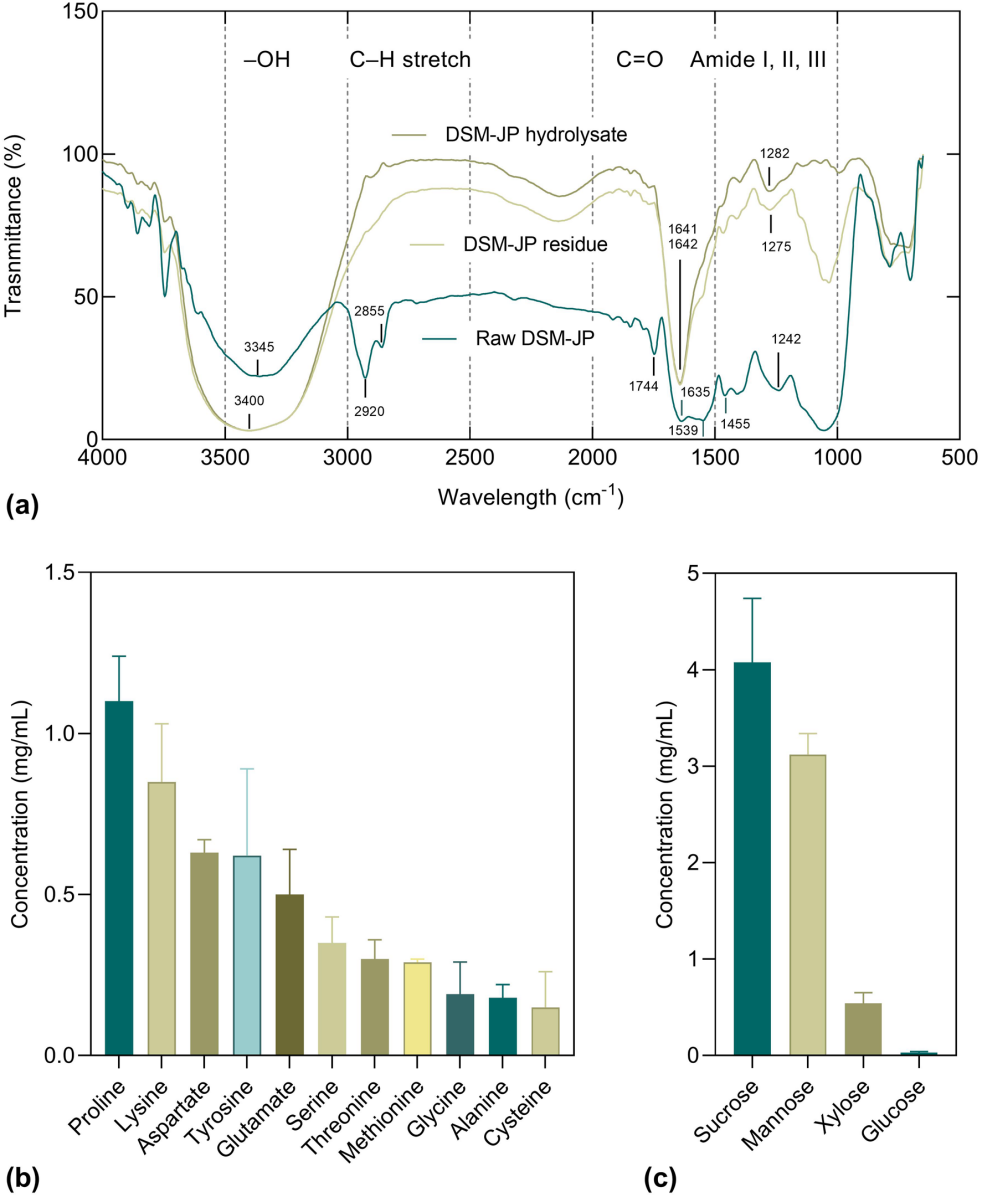
Changes in the chemical structure of hydrolyzed DSM-JP: (**a**) FTIR spectra of raw, residue, and hydrolysate of DSM-JP; (**b**) amino acid and (**c**) sugar profiles of DSM-JP hydrolysate. Mean data of triplicate experiments were presented with error bars represent standard deviations. The graphs were visualized in GraphPad PRISM software (v 8.02; GraphPad, Inc., MN, USA; www.graphpad.com).

This section delves deeper into the structural and chemical changes made to the DSM-JP biomass and hydrolysate produced. An FTIR spectroscopy analysis was performed on the raw, residue, and hydrolysate of DSM-JP as illustrated in Fig. 3a. In comparison to the DSM-JP residue, the raw biomass had a low transmittance, indicating the structural rigor of the original biomass. Strong bonds, such as hydrogen bonds, disulfide bonds, and peptide bonds, reinforce the protein and carbohydrate structure of the raw DSM-JP. Since the raw DSM-JP is densely packed, it obstructs light transmission in the FTIR analysis. The high transmittance of the residue and hydrolysate, on the other hand, indicates that the treatments had caused severe structural damage to the original biomass^32^.

The strengths of the vibrating bonds and the masses of the vibrating atoms were shown to have the greatest impact on the vibrational frequencies of the bonds^44^. According to the protein conformational reports^45,46^, the common band at 1600–1700 cm^−1^ was frequently used as a protein indicator, which was associated with amide I presence (primarily C=O stretching vibration of the polypeptide backbone, combined with C–N stretching, C–C–N deformation, and N–H bending modes in plants). As shown in the FTIR spectra, the peak of amide I in DSM-JP residue and hydrolysate was shifted from 1635 to 1641 cm^−1^ and 1642 cm^−1^, respectively, and the intensity increased from 6.45 to 19.19% and 19.79%, respectively, indicating an increase in the length of the peptide dipole moment and demonstrating polypeptide backbone motion exerted by the MAH treatment^47^.

This finding is consistent with that of Tian et al.^48^ who reported a similar band shift of the aqueous layer of soybean protein hydrolysate (ASPH) spectrum after an extended duration of ultrasound treatments. Further characterization revealed that the secondary structures of the ASPH were significantly altered where the *β*-sheet content was increased but the *α*-helix and *β*-turn content was reduced as compared to the control. This might explain the shift in the amide I absorptions in this study since this frequency region is a sensitive protein fingerprint to indicate changes of the protein secondary structures e.g., *α*-helix, *β*-turn, *β*-sheet, and random coil^49^. The breakdown of hydrogen bonds inside peptide molecules may be responsible for the alterations in protein aggregates of DSM-JP. According to Guzmán-Ortiz et al.^50^, high-temperature treatments trigger alterations in secondary, tertiary, and quaternary protein structure, exposing polypeptides and open internal peptide bonds, making them more easily hydrolyzed.

Similarly, both amide II (approx. 1500–1600 cm^−1^) and amide III bands (approx. 1100–1500 cm^−1^), which correlate to N–H bending and chemical groups in protein side chains^51,52^, exhibited frequency alterations as compared to the raw DSM-JP. The amide II band of the raw DSM-JP was detected at 1539 cm^−1^, but the corresponding bands were not detectable for both residue and hydrolysate. Whereas the amide III band for both residue and hydrolysate showed medium absorptions at 1275 cm^−1^ and 1282 cm^−1^, respectively, which notably shifted from 1242 cm^−1^ recorded in the raw DSM-JP spectrum. These differences reflected a modification in the secondary structure of DSM-JP proteins, which concur with the frequency vibrations demonstrated in the amide I band resulted from the MAH treatment.

The band in the high-energy region is commonly attributed to a large abundance of O–H groups in carbohydrates and lignin^53^. The most prominent bands at 3000–3500 cm^−1^, which correspond to hydrogen-bonded O–H stretching of hydroxyl groups deriving predominantly from cellulose and hemicelluloses, were observed in all spectra^39,54^. The broad, strong peak of the raw DSM-JP at 3345 cm^−1^ was observed shifted to 3400 cm^−1^ for both DSM-JP residue and hydrolysate. In addition, the presence of carbonyl bands at 1630–1650 cm^−1^ and 1740–1760 cm^−1^ respectively, in the raw DSM-JP, indicate the presence of free and esterified carboxyl groups prior to hydrolysis^55^. The absence of shoulder peak at 1744 cm^−1^ after the MAH treatment suggests that the acetyl and uronic ester groups of hemicelluloses, as well as the ester bond of lignin, might be broken down during the process. This is confirmed by the absence of absorption bands at 1455 and 1539 cm^−1^ which indicates the lack of C=C aromatic ring of lignin^25^. Therefore, the breakdown of non-soluble polysaccharides of DSM-JP could be a major factor, causing the release of abundant soluble sugars in the hydrolysate produced.

The free amino acid and sugar profiles of the hydrolysate are presented in Fig. 3b, c, respectively. The composition of each amino acid ranged from the highest at 1.10 mg/mL (proline) to the lowest at 0.15 mg/mL (cysteine). The amino acid yield (5.14 mg/mL) in this investigation was much lower as compared to the enzymatic hydrolysis (endopeptidase) of DSM demonstrated by Liu et al.^21^ who obtained more than 25.6 mg/mL. The differences between these findings are most likely due to the possibility that these amino acids were still present in the form of intact protein and peptides in the residue, or that the amino acids were further degraded to low molecular weight carboxylic acids such as formic acids, acetic acids, propionic acids, etc. as a result of the excessive microwave irradiation^21,37^.

The sugar profile as illustrated in Fig. 3c shows that the DSM-JP hydrolysate was predominantly comprised of sucrose (4.08 mg/mL), followed by mannose (3.12 mg/mL), xylose (0.54 mg/mL), and glucose (0.03 mg/mL). The ample composition of reducing sugars such as mannose and xylose in the hydrolysate suggested that they might be produced from the hemicellulose breakdown of biomass which was consistent with the previous FTIR spectra analysis. The scarcity of glucose content, as well as the other undetectable monosaccharides e.g., fructose and galactose, in the hydrolysate, could be ascribed to the occurrence of thermal-alkaline degradation of reducing sugars during the MAH treatment^56^. The condensation of reducing sugar (the early stage of the Maillard reaction) such as glucose, with a free amino group e.g., lysine, results in protein glycation reaction, producing the Amadori products which eventually degraded into furfurals, reductones, and fragmentation products i.e., carbonyl and hydroxycarbonyl compounds^57^.

### Biocompatibility of DSM-JP hydrolysate and production of SJMo-Alg bead fertilizer

**Figure 4:**
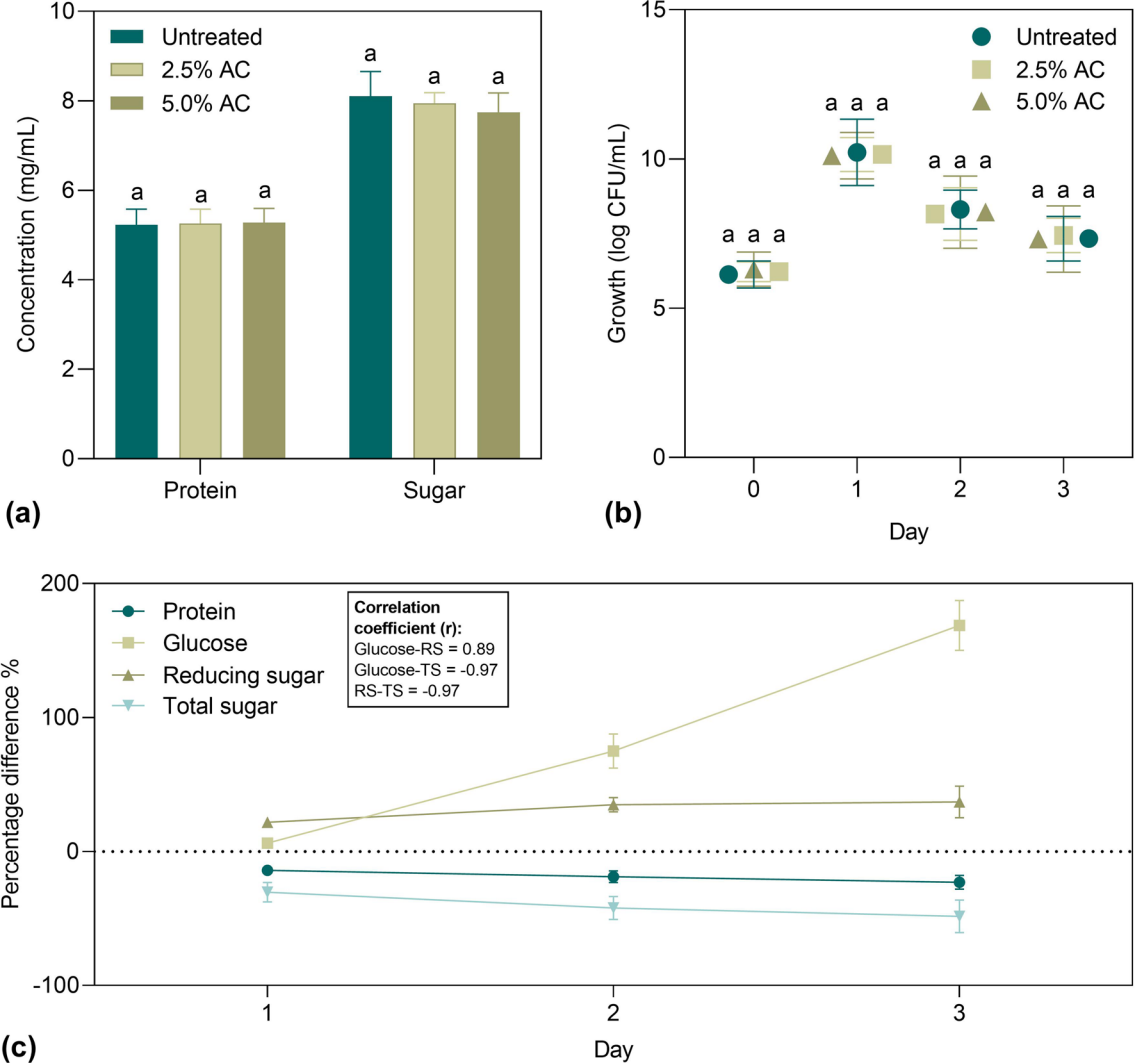
Biocompatibility assay of DSM-JP hydrolysate: (**a**) detoxification of hydrolysate using activated carbon (AC); (**b**) Growth of strain 40a in a raw and a detoxified hydrolysate media (**c**). nutrient utilization dynamics during 3-day growth of strain 40a. Data represent mean values ± standard deviations (n = 3). Different letters indicate statistically significant differences between factors (two-way ANOVA + Tukey multiple comparisons test at *P* < 0.05). The correlation coefficient was calculated by Pearson correlation. The graphs were visualized in GraphPad PRISM software (v 8.02; GraphPad, Inc., MN, USA; www.graphpad.com).

The formation of inhibitory compounds/ by-products such as acetic acid, hydroxy acids, dicarboxylic acids, and phenolic compounds has been linked to the use of mild alkaline treatment in the removal of resistant lignin and hemicellulose from agro-waste^31^. As a precautionary measure, the DSM-JP hydrolysate was primarily detoxified with activated carbon to remove the possible inhibitory compounds formed during the MAH treatment prior to use as growth media. The effects of detoxification on the soluble protein and sugar content in the hydrolysate were illustrated in Fig. 4.

According to the Fig. 4a, even with varying percentages of AC (2.5% and 5% w/v) employed, both protein and sugar concentrations in the hydrolysate were minimally impacted (*P* > 0.05). A marginal sugar loss of 2% and 4.4% were detected in 2.5% and 5% AC treatment, respectively. These results are consistent with the data obtained by Zhang et al.^58^. In addition, the biocompatibility assay of DSM-JP hydrolysate on strain 40a as presented in Fig. 4b showed that both raw and detoxified hydrolysate could support the growth of strain 40a with no significant difference (*P* > 0.05) observed on cell counts during the 3-day incubation. As a result, it can be concluded that the raw DSM-JP hydrolysate requires no detoxification and can be utilized directly as a nutritional medium additive for strain 40.

The nutrient utilization dynamic (protein, glucose, reducing sugar, and total sugar) during the growth of strain 40a in the neutralized DSM-JP hydrolysate was further evaluated as illustrated in Fig. 4c. Contrary to expectations, the protein content was gradually declined while the glucose content was dramatically inclined. These findings show that strain 40a only used protein hydrolysate sparingly for cell development and rarely utilized glucose. This counterintuitive outcome differs from our previous observations in which strain 40a grew optimally with the supplementation of DSM protein and glucose as the most favourable nitrogen and carbon source, respectively^59^. Surprisingly, the total sugar gradually decreased until day 3, reaching a high of 48% reduction, whereas glucose and reducing sugar increased up to 169% and 37%, respectively. This result implies that strain 40a might favour non-reducing sugars (e.g., sucrose) over monomeric sugars when DSM-JP hydrolysate was used as growth media.

The strong, inverse correlation (r =  − 0.97) between the reduction of total sugar and the increase in glucose and reducing sugar implies that their accumulation could be due to the breakdown of non-reducing sugar. Due to the abundance of sucrose in the hydrolysate, strain 40a tends to consume sucrose over other reducing sugars e.g., mannose and xylose. The breakdown of sucrose into its constituent monosaccharides, glucose, and fructose might explain the increasing pattern of both glucose and reducing sugar levels in the medium. Beisel and Afroz^60^ described that bacterial cells, in general, are assumed to form a preference hierarchy as they begin with nutrients that are more quickly catabolized before moving on to others. Bacteria are assumed to abandon their pickiness and eat whatever is available when only poor nutrients are accessible, or the nutrients are present in low amounts.

**Figure 5:**
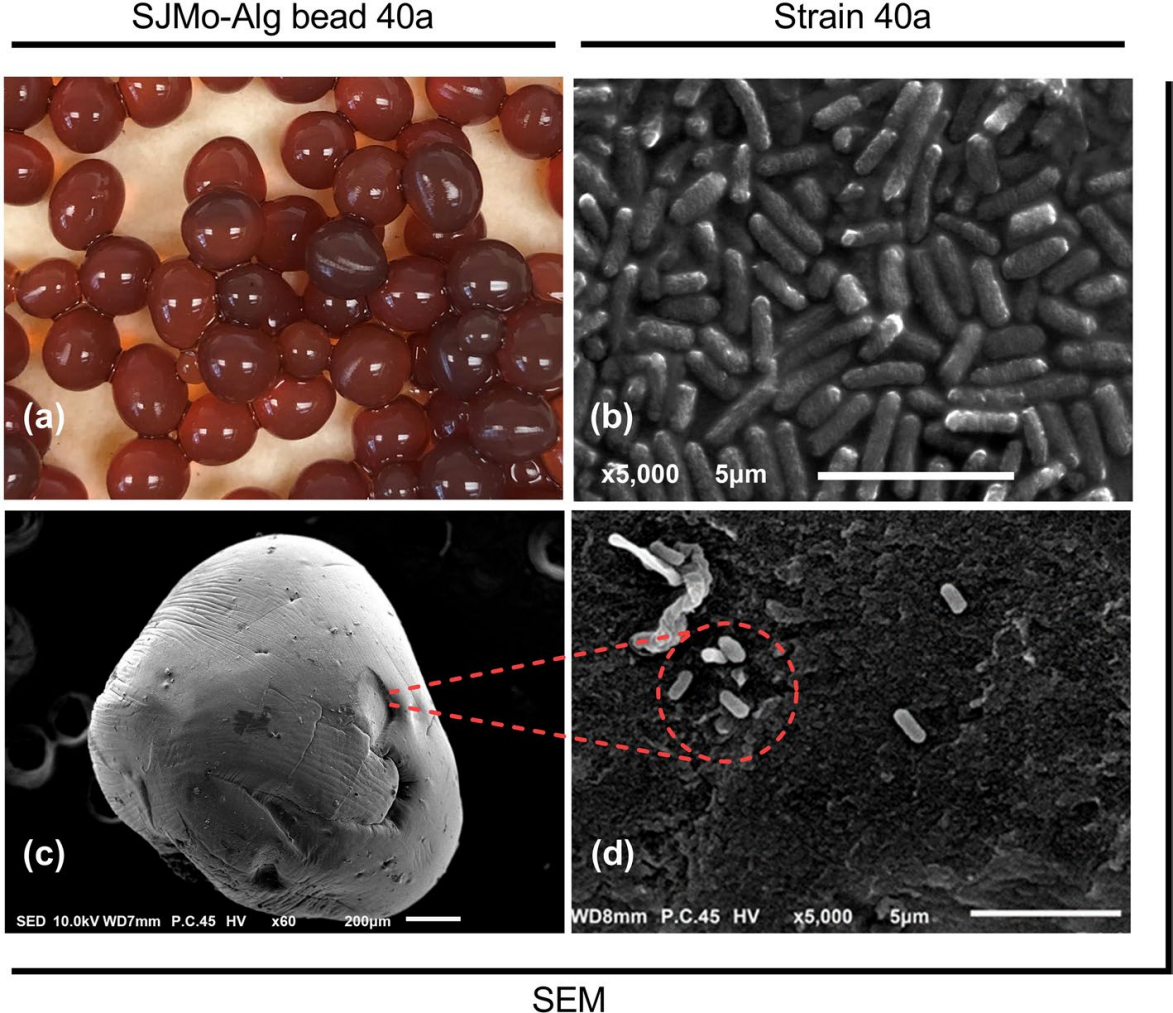
The visual image of SJMo-Alg bead 40a biofertilizer: (**a**) representative image of the freshly extruded bead; (**b**) SEM image of free cells of strain 40a; (**c**) SEM image of a single bead; (**d**) high magnification SEM image of encapsulated strain 40a on the surface of SJMo-Alg bead 40a.

The DSM-JP hydrolysate was then transformed into an alginate bead through encapsulation of strain 40a using sodium alginate and molasses (SJMo-Alg bead 40a). Molasses was incorporated in the bead formulation as a polycationic supplementary nutrient and mineral supply for strain 40a, as well as to improve the porosity of sodium alginate^9^. The representative image of SJMo-Alg bead 40a and the SEM image of a single bead are presented in Fig. 5. The beads appeared dark brownish red, mostly due to the colour combination of DSM-JP hydrolysate and molasses, shiny surface, almost spherical, and about 3–5 mm in size. Prior to further examination, the beads were aseptically transferred and stored in a refrigerator in a tightly sealed 1 L Schott bottle.

### Performance of SJMo-Alg bead 40a in P and K solubilization

**Figure 6:**
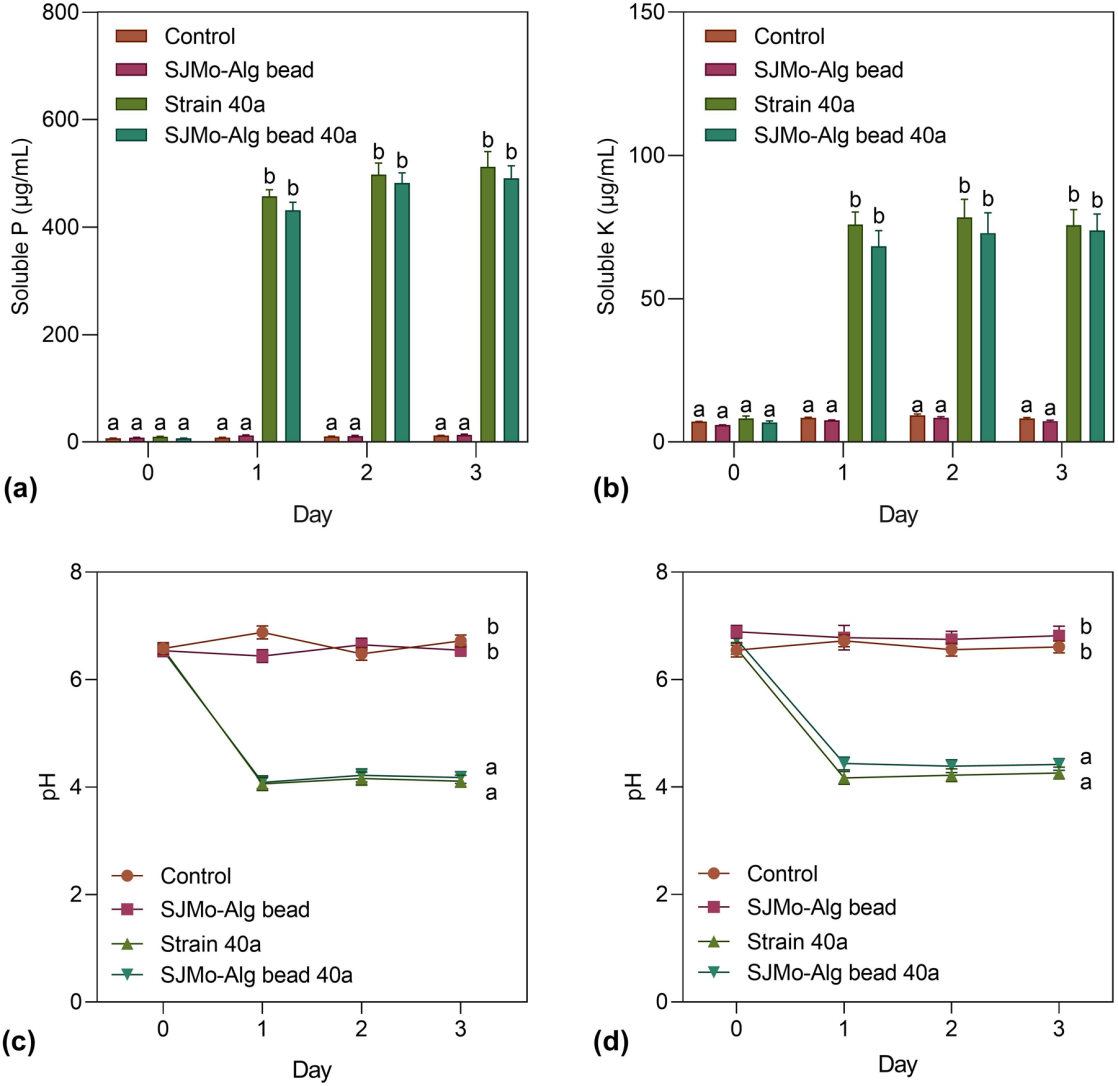
Comparison of P and K solubilization activity of free cells and encapsulated cells of strain 40a (SJMo-Alg bead 40a). Data represent mean values ± standard deviations (n = 3). Different letters indicate statistically significant differences between factors (two-way ANOVA + Tukey multiple comparisons test at *P* < 0.05). The graphs were visualized in GraphPad PRISM software (v 8.02; GraphPad, Inc., MN, USA; www.graphpad.com).

The performance of SJMo-Alg bead 40a in P and K solubilization was compared with the free cells of strain 40a (Fig. 6). As presented in the figure, the P release achieved the highest point after day 3, which was 512.16 µg/mL and 490.81 µg/mL for free cell and encapsulated 40a, respectively. Both free cell and encapsulated 40a demonstrated no significant difference (*P* > 0.05) in the solubilized P and pH in the NBRIP media. Likewise, the solubilized K and pH in the Aleksandrov media of both free cell and encapsulated 40a showed no significant difference (*P* > 0.05) during the 3-day incubation. The highest solubilized K achieved was 78.34 µg/mL and 73.88 µg/mL for free cell and encapsulated 40a, respectively. Both NBRIP and Aleksandrov media turned to acidic pH on day 1 and were maintained around pH 4 until the end of the experiment. These results were coherent with those observed in our prior research on P and K solubilization activities of strain 40a^5^. These results provide further support for the hypothesis that the alginate encapsulation does not deteriorate or compromise the biological cellular activities of strain 40a, hence suitable as a biofertilizer candidate particularly for soil available P and K amelioration.

## Materials and methods

### Materials preparation and characterization

DSM was purchased from D’syira Enterprise Sdn. Bhd., Serdang, Selangor, Malaysia, while JP was collected from a night market in Sri Serdang, Selangor, Malaysia. Both raw samples were air-dried outside during the day under shade for 2 days, followed by oven-drying (Protech, FDD-720, Cincinnati, Ohio) at 70 °C for 24 h. The dried samples were ground into a coarse powdery texture using a mechanical Waring blender (8010S/G, PA, USA). Then, they were strained using a 2 mm Gilson sieve and kept in a tight container separately at room temperature prior to analysis. The nutrient composition of the treated samples was characterized as follows: protein content using the Kjeldahl method (Gerhards Vapodest 50s, Hillerød, Denmark); total fat using the Soxhlet extraction method (Gerhards Soxhterm, Hillerød, Denmark); moisture by oven-drying; ash content by furnace incineration^61^ (Carbolite, Derbyshire, UK); total carbohydrate by calculation according to Pomeranz and Meloan^62^.

### Microwave-alkaline hydrolysis (MAH)

MAH treatment was carried out in a 32 L domestic microwave oven (Samsung, MS32J5133GM/SM, Seoul, South Korea) with an operating frequency of 2450 MHz. Throughout the whole experiment, the hydrolysis procedure of Cheong et al*.*^32^ was adopted with some adjustments. About 2 g of 1:1 (w/w) ratio of DSM to JP was mixed with 100 mL of NaOH solution with varying concentrations of 0.04 to 0.11 M. The microwave was set at a power level of 30 with a varying residence time of 2 to 11 min. The hydrolysate produced was collected by vacuum filtration and deionized water was added to bring the hydrolysate to the original volume.

### Optimization of MAH by response surface methodology (RSM)

The MAH treatment was optimized through response surface methodology (RSM) using CCD. The regression analysis of the variables was performed in Design of Expert (DOE) software (v 11; Stat-Ease, Inc., MN, USA; www.statease.com). The factors with a *P*-value less than 0.05 were regarded to have a significant impact on the response in the analysis. The experimental design in this study involved 13 trials and 5 replicates at the center point. Assuming a second-order polynomial function with linear, quadratic, and interaction effects, the system's function was determined as follows:

3 $$Y = \beta_{o} + \mathop \sum \limits_{i = 1}^{n} \beta_{i} X_{i} + \mathop \sum \limits_{i = 1}^{n} \beta_{ii} X_{i}^{2} + \mathop \sum \limits_{i}^{n} \mathop \sum \limits_{j}^{n} \beta_{ij} X_{i} X_{j}$$

where Y is the predicted result, β_0_ (offset term), β_i_ (linear effect), β_ii_ (quadratic effect), and β_ij_ (interaction effect) are constant coefficients, and X shows the coded level of the independent variable. After all the response data had been inserted, DOE software generated the regression analysis of variance including the graphical response surface plots. The optimal MAH treatment parameters for maximum protein and sugar yields were estimated by statistical analysis.

### Estimation of protein and sugar content

The soluble protein and soluble sugar content recovered from all the experimental trials was determined by the Bradford protein assay^63^ and the phenol–sulphuric acid method^64^, respectively. Bovine serum albumin (3 mg/mL) served as the protein standard while glucose (1 mg/mL) served as the sugar standard. The absorbance was measured using a UV–VIS spectrophotometer (Secomam Uviline 9400, France) at 595 nm (protein) and 470 nm (sugar). The protein and sugar recovery from the MAH treatment of the DSM-JP cocktail was calculated as follows:

4 $$Protein\;recovery{:}\quad g{/}g\;total\;protein = \left( {P \times V} \right)/1000\;P_{t}$$

5 $$g{/}g\;dry\;mass = \left( {P \times V} \right)/1000\;W$$

6 $$Sugar\;recovery{:}\quad g{/}g\;total\;sugar = \left( {S \times V} \right)/1000\;S_{t}$$

7 $$g{/}g\;dry\;mass = \left( {S \times V} \right)/1000\;W$$

where P is the soluble protein and S is the soluble sugar in DSM-JP hydrolysate (mg/mL); V is the sample volume (mL); P_t_ is the total protein content of DSM-JP (g); S_t_ is the total sugar content of DSM-JP (g); W is the initial weight of DSM-JP biomass (g).

### Fourier transform infrared (FTIR) spectroscopy

The functional groups present in the test samples were identified via FTIR analysis. This analysis was conducted in the attenuated reflectance mode (Spectrum 100, PerkinElmer, MA, USA). A total of 16 scans were completed at a resolution of 4 cm^−1^ from 4000 to 650 cm^−1^ for spectral manipulation of spectra and analysis.

### Amino acid and sugar profiling

The free amino acid compositions in DSM-JP hydrolysates were determined by AccQ Fluor Reagent derivatization RP-HPLC (Waters 2695, Waters Corporation Milford, MA, USA). The HPLC system was equipped with a Waters 2475 fluorescence detector and an AccQ Tag Column (3.9 × 150 mm). The mobile phase was AccQ Tag Eluent A concentrate, and AccQ Tag B, or 60% acetonitrile. The derivatization of the samples and the standard amino acids (i.e., glutamic acid, aspartic acid, serine, histidine, glycine, methionine, arginine, alanine, threonine, proline, cysteine, tyrosine, valine, isoleucine, lysine, leucine, and phenylalanine) determination process were recommended by Welch Materials (http://www.welch-us.com). Sugar profile was determined using Waters Alliance 2695 HPLC Separations Module (ELS 2410) System (USA) with Waters 2410 RI detector against standards of 1 mg/mL sucrose, glucose, fructose, galactose, mannose solution (Sigma).

### Detoxification of hydrolysate using activated carbon

Concentrated NaOH (50% w/v) was added dropwise to the DSM-JP hydrolysate to adjust the pH to 6.5–7.0. The detoxification procedure was performed using AC according to Zhang et al*.*^58^. Exactly 100 mL of hydrolysate was treated with 2.5% or 5.0% (w/v) of AC in a 250 mL Erlenmeyer flask with a ground-glass stopper. The flask was placed on an orbital shaker and mixed at 200 rpm for 2 h. The treated hydrolysate was collected after separating the AC by vacuum filtration. Deionized water was added to bring the hydrolysate to the original volume. The change in protein and sugar content was assessed using the methods outlined previously.

### Biocompatibility of DSM-JP hydrolysate with strain 40a

*E. hormaechei* 40a was obtained from the bacterial culture collection at Research Laboratory 1.6, Bioprocessing and Biomanufacturing Research Centre, Universiti Putra Malaysia. To obtain a pure single colony, strain 40a was subcultured on a nutrient agar plate regularly. A fresh overnight culture (1 mL of ~ 10 log CFU/mL) was inoculated in a 150 mL Erlenmeyer flask containing 50 mL of raw or treated hydrolysate at neutral pH (6.8–7.0), and the bacteria was cultured at 37 °C for 3 days while shaking at 200 rpm. Culture sampling was conducted every 24 h for the determination of viable cell count by serial dilution with 0.1% (v/v) NaCl solution. Strain 40a was enumerated on nutrient agar for 24 h at 37 °C and expressed as log CFU/mL. During the incubation, the nutrient utilization dynamic of strain 40a was evaluated based on the daily percentage difference (against the initial value) of protein, glucose, reducing sugar, and total sugar content in the media. About 1 mL of cell-free supernatant was subjected to protein and total sugar assay using methods outlined previously, glucose assay using glucose oxidase/peroxidase (GOPOD) kit (Megazyme Co., Wicklow, Ireland), and reducing sugar assay using 3,5-dinitrosalicylic acid (DNS) against 1 mg/mL glucose standard^65^.

### Bioencapsulation using molasses-alginate matrix and morphology observation

Sodium alginate solution was used to establish a stable cell encapsulation and extrusion process^66^. After cell washing, a ~ 10 log CFU/mL culture pellet was suspended in 50 mL DSM-JP hydrolysate (pH 6.5–7.0) and combined with 50 mL of 3% (w/v) homogenized sodium alginate solution (sterilized at 121 °C for 15 min). The cell suspension was then extruded through a silicone tube (internal diameter 3 mm) by using a microtube pump MP-3 N (Eyela, Japan) into a sterile 100 mL of 3% (w/v) CaCl_2_ (premixed with 1.5% molasses) with a delivery rate of 30 beads/min. The beads were then left to gel for 30 min before being washed with a 0.1% (v/v) sterile NaCl solution. The uninoculated control beads were prepared similarly but without the culture pellet.

The morphology observation of free cells of strain 40a and the alginate beads were performed by scanning electron microscope (SEM) (JSM-IT100 InTouchScope™, JEOL, Tokyo, Japan). An overnight culture of free cells of strain 40a was harvested via centrifugation at 7000 rpm for 5 min at 4 °C, and the cell pellets were washed with a 0.1% (v/v) sterile NaCl solution. Samples were mixed with 4% glutaraldehyde in a separate microcentrifuge tube or vial and fixed for 2 days at 4 °C. Samples were washed three times with 0.1 M sodium cacodylate buffer of 30 min soaking each. Post-fixation of samples was performed using 1% osmium tetroxide for 2 h at 4 °C prior to rewashing three times with 0.1 M sodium cacodylate buffer. Samples were dehydrated with a series of acetone washing (35% to 100% concentration) for 1 h incubation time each. A critical point dryer (Autosamdri®-815) was used for final dehydration for about 1 h before mounting onto the stub. Dehydrated samples were coated with gold using a sputter coater and transferred to a slide for viewing.

### P and K solubilization assay

To measure the performance of cell-encapsulated beads for P and K solubilization activity, it was compared with the free cell of strain 40a using the methods described by Roslan et al.^5^. P solubilization assay was performed using a modified National Botanical Research Institute’s Phosphate (NBRIP) medium which contained (per Liter): 10 g glucose; 5 g Ca_3_(PO_4_)_2_; 5 g MgCl_2_.6H_2_O; 0.25 g MgSO_4_.7H_2_O; 0.2 g KCl, 0.1 g (NH_4_)0.2SO_4_ and 10 mL of 0.5% bromothymol blue (dissolved in 0.2 N KOH). K solubilization assay was performed using a modified Aleksandrov medium (Himedia) mixed with 0.018 g/L phenol red dye. A fresh overnight culture (1 mL of ~ 10 log CFU/mL) or 1 g of cell-encapsulated beads was inoculated in a 150 mL Erlenmeyer flask containing 50 mL NBRIP or Aleksandrov media and incubated at 37 °C for 3 days while shaking at 200 rpm. Sampling was done every 24 h where 1 mL of cell-free aliquot was taken to measure the pH and the soluble P and K of the media. The soluble P was determined by the yellow phospho-molybdo-vanadate colorimetry^67^ using UV–VIS spectrophotometer at 430 nm, while the soluble K was determined using atomic absorption spectrometer (AAS) with the flame air-C_2_H_2_ at the wavelength of 766.5 nm.

### Statistical analysis

All statistical analyses in RSM were performed using DOE software, while the rest of the experiments i.e., student’s *t*-test, Pearson correlation, and two-way ANOVA (with Tukey multiple comparisons test) were performed using GraphPad PRISM software (v 8.02; GraphPad, Inc., MN, USA; www.graphpad.com). A *P*-value < 0.05 was considered statistically significant.

## Conclusion

The MAH treatment was able to recover a high percentage of native proteins and sugars from the DSM-JP biomass cocktail and produced a nutrient-dense hydrolysate under the following conditions: DSM-JP biomass, 2 g; NaOH concentration, 0.084 M (100 mL); residence time, 8.7 min; microwave power-level, 300 W. FTIR analysis revealed that the chemical structure and composition of DSM-JP changed after the full processing of optimized thermochemical treatment. Detoxification of raw DSM-JP hydrolysate using AC showed neither significant changes in protein and sugar content, nor did it affect the biocompatibility of strain 40a to grow in it, as compared to the raw hydrolysate. The performance in P and K solubilization by the encapsulated strain 40a (SJMo-Alg bead 40a) was remarkably maintained and comparable to the free cell counterpart. These findings indicate the potential of SJMo-Alg bead 40a as a biofertilizer candidate particularly for soil available P and K amelioration. Future prospects of this investigation should include the performance of SJMo-Alg bead 40a in promoting plant growth as well as the P and K acquisition.

## Supplementary Information

Supplementary Table S1.
