# Supplementary material for: Bioprospecting microwave-alkaline hydrolysate cocktail of defatted soybean meal and jackfruit peel biomass as carrier additive of molasses-alginate-bead biofertilizer
Source: Sci Rep. 2022 Jan 7;12:254. doi: 10.1038/s41598-021-02170-w (PMC8742054; doi:10.1038/s41598-021-02170-w)
Supplement: Supplementary file 1 — Supplementary Table S1. [file 41598_2021_2170_MOESM1_ESM.docx]

**Table S1.**  Chemical characteristics of defatted soybean meal and jackfruit peel.

| **Characteristic** | **Defatted soybean meal** | **Jackfruit peel** |
| --- | --- | --- |
| pH (H_2_O) | 6.21 ± 0.42 | 6.32 ± 0.39 |
| Moisture content (%) | 10.23 ± 1.95 | 61.73 ± 0.73 |
| **Soluble sugar^a^ (mg/g)** | **dmb** | **dmb** |
| Sucrose | 81.21 ± 4.33 | 11.17 ± 1.10 |
| Glucose | nd | 90.27 ± 1.37 |
| Fructose | nd | 186.48 ± 0.82 |
| **Elemental analysis** |  |  |
| **Macroelement (mg/g dmb)** |  |  |
| Carbon, C^b^ | 421.18 ± 18.7 | 427.69 ± 1.40 |
| Nitrogen, N^b^ | 78.17 ± 0.8 | 11.31 ± 0.80 |
| Sulphur, S^b^ | 3.29 ± 0.6 | 0.89 ± 0.11 |
| Phosphorus, P^c^ | 2.12 ± 0.27 | 0.13 ± 0.03 |
| Potassium, K^d^ | 23.34 ± 0.18 | 45.1 ± 8.42 |
| Calcium, Ca^d^ | 4.05 ± 0.14 | 4.55 ± 1.55 |
| Magnesium, Mg^d^ | 3.68 ± 0.42 | 2.58 ± 0.19 |
| Sodium, Na^d^ | 2.6 ± 0.16 | 13.22 ± 0.78 |
| **Microelement (µg/g dmb)** |  |  |
| Iron, Fe^d^ | 0.21 ± 0.02 | 131.4 ± 2.9 |
| Boron, B^d^ | 0.12 ± 0.01 | 318.5 ± 33.8 |
| Manganese, Mn^d^ | 0.04 ± 0.02 | 37.1 ± 12.51 |
| Molybdenum, Mo^d^ | 0.01 ± 0.01 | 5.3 ± 1.21 |
| Zinc, Zn^d^ | 0.08 ± 0.02 | nd |

Value is expressed in mean ± standard deviation (n = 3 biological replicates)

nd—not detected, wmb—wet matter basis, dmb—dry matter basis.

^a^ determined by HPLC analysis.

^b^ determined by CNS elemental analysis.

^c^ determined by the yellow phospho-molybdo-vanadate colorimetry [27].

^d^ determined by ICP-OES analysis.
